# Supplementary material for: Live-cell single particle tracking of PRC1 reveals a highly dynamic system with low target site occupancy
Source: Nat Commun. 2021 Feb 9;12:887. doi: 10.1038/s41467-021-21130-6 (PMC7873255; doi:10.1038/s41467-021-21130-6)
Supplement: Supplementary file 1 — Supplementary Information [file 41467_2021_21130_MOESM1_ESM.pdf]

**Supplementary Information**

# Live-cell single particle tracking of PRC1 reveals a highly dynamic system with low target site occupancy

Huseyin and Klose

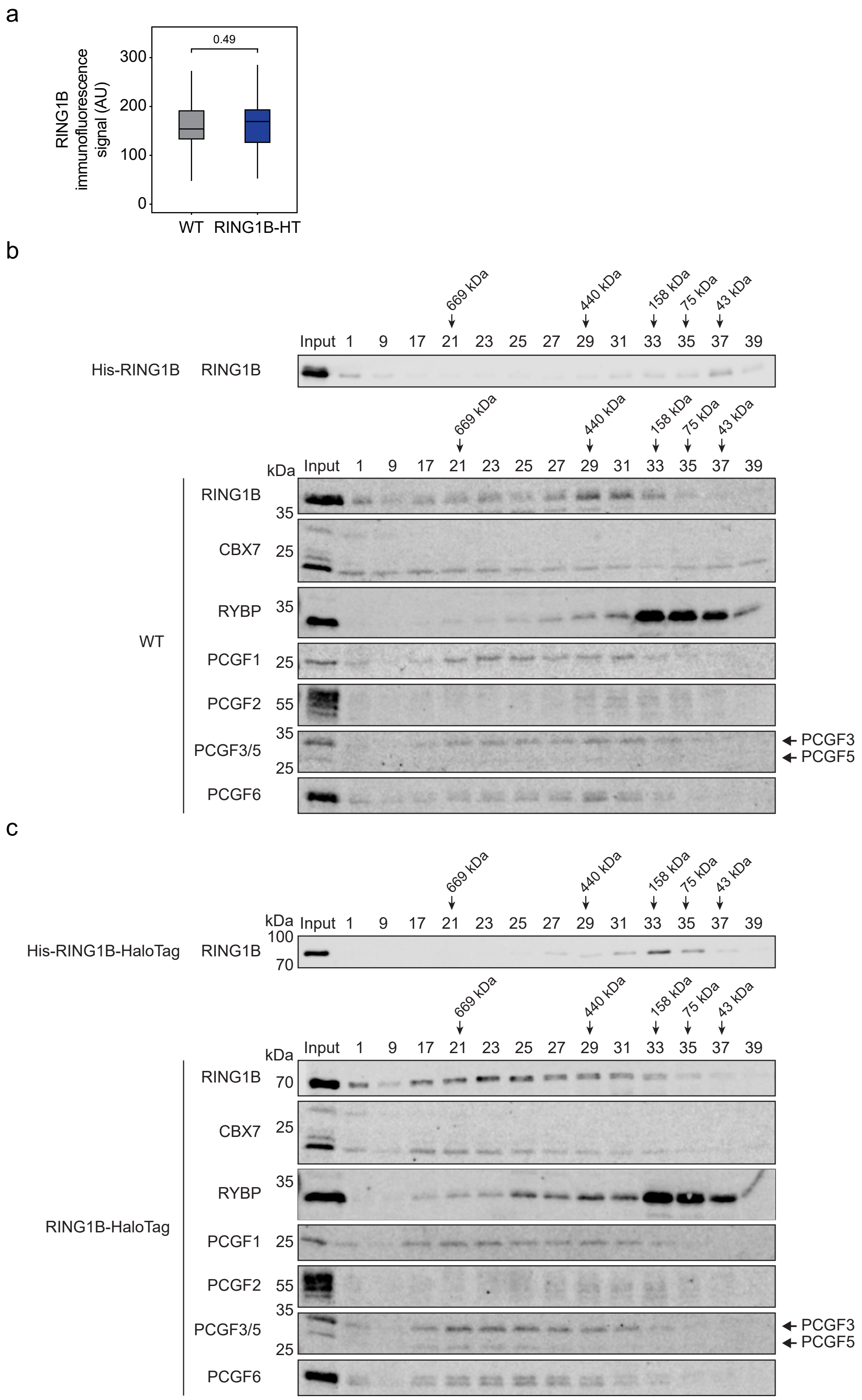


Supplementary Fig. 1

**Supplementary Fig. 1 – RING1B-HaloTag is expressed and forms complexes similarly to wild type RING1B.**

**a** A box plot comparing the nuclear RING1B immunofluorescence signal in mixed untagged (WT, grey) and RING1B-HT ESCs (blue). This illustrates that the levels of RING1B are equivalent in wild type and RING1B-HT cells. *n* = 41 cells per cell line from 3 experiments. Boxes represent the interquartile range (IQR), the middle line corresponds to the median, and whiskers extend to the largest and smallest values no more than 1.5 x IQR from the box. Values outside of this range are not plotted, but are included in all analyses. Source data are provided as a Source Data file.

**b** Size exclusion chromatography analysis of recombinant RING1B and nuclear extract from wild type ESCs probed by western blot with the indicated antibodies. Recombinant RING1B indicates the size of monomeric non-complexed RING1B. The majority of RING1B is found in high molecular weight PRC1 complexes. *n* = 1 independent replicate. Source data are provided as a Source Data file.

**c** Size exclusion chromatography analysis of recombinant RING1B-HaloTag and nuclear extract from RING1B-HaloTag ESCs probed by western blot with the indicated antibodies. This demonstrates that RING1B-HaloTag incorporates into PRC1 complexes with the same efficiency as endogenous RING1B. RING1B-HaloTag and its associated complexes elute in higher molecular weight fractions due the addition of the 34 kDa HaloTag. *n* = 1 independent replicate. Source data are provided as a Source Data file.

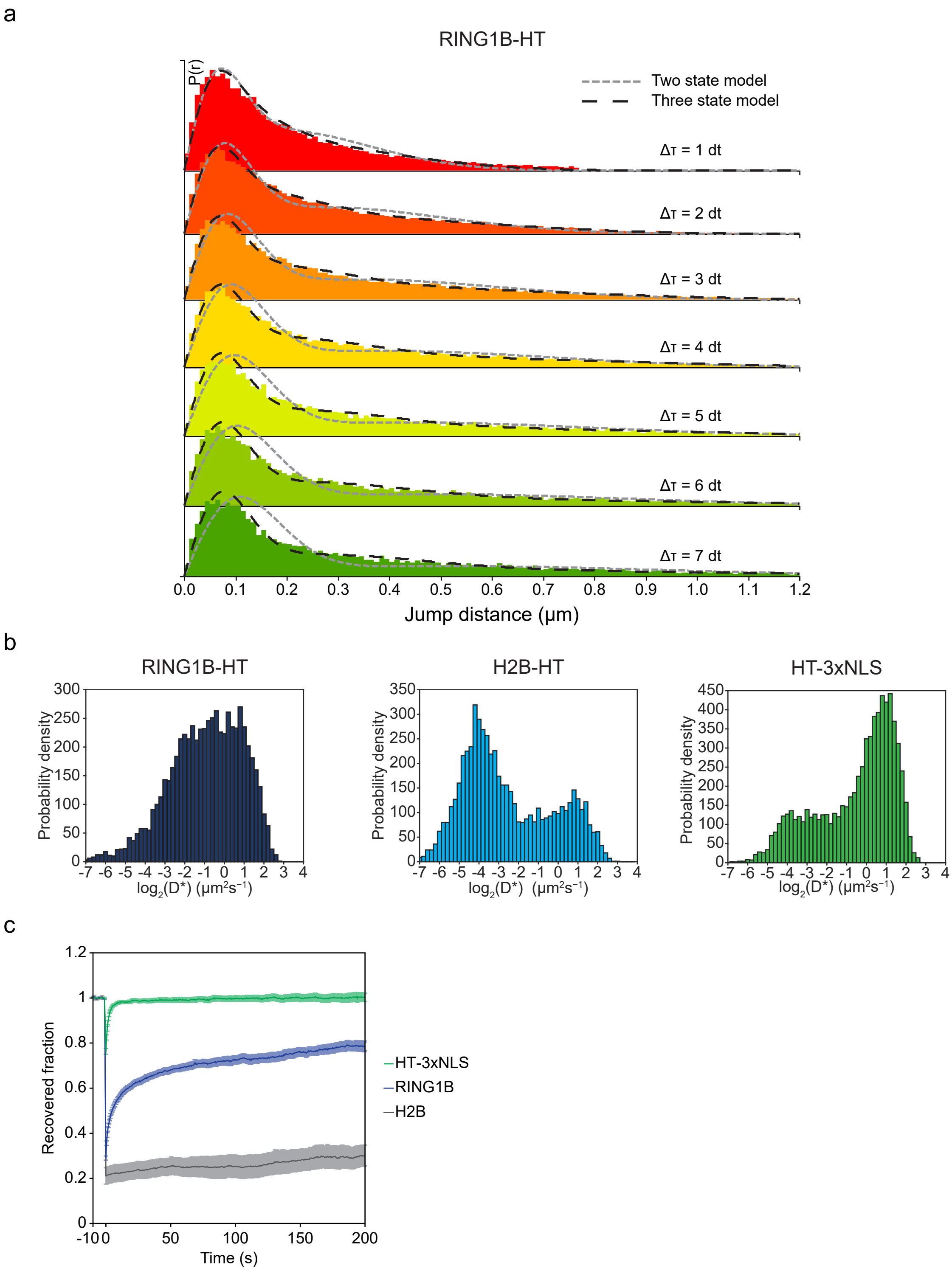


Supplementary Fig. 2

**Supplementary Fig. 2 – Live cell imaging and single particle tracking allows for measurement of RING1B diffusion.**

**a** Pooled jump length histograms from all movies from a single RING1B SPT experiment overlaid with two-state and three-state models (dashed lines) produced by Spot-On. Histograms show the lengths of first four steps for all tracks starting from the indicated $\Delta t$.

**b** Example histograms of apparent diffusion coefficients ($D^*$, $\mu m^3$ $s^{-1}$) calculated from tracking experiments for individual RING1B (dark blue), H2B (light blue) and HaloTag-3xNLS (green) molecules. Tracks analysed are from a single representative experiment (total $n$ = 3) pooling at least 10 movies per protein. Source data are provided as a Source Data file.

**c** FRAP recovery curves for HaloTag-3xNLS (green), RING1B (dark blue) and H2B (light blue). The recovered fraction was measured relative to initial fluorescence intensity, corrected using an unbleached region. Error bars denote SEM for $n$ > 10 cells per condition from 2 experiments. Source data are provided as a Source Data file.

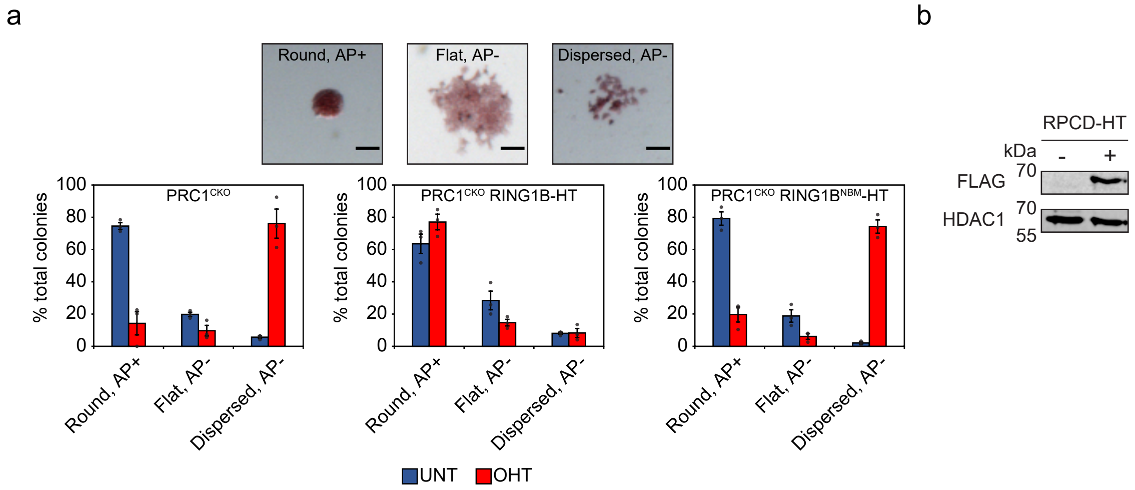


Supplementary Fig. 3

**Supplementary Fig. 3 – A nucleosome binding mutant of RING1B is insufficient to rescue defects in morphology and pluripotency caused by RING1B deletion.**

- **a** Alkaline phosphatase (AP) staining analysis of PRC1$^{CKO}$ ESC colonies with (red) and without (blue) 72 hr OHT treatment to assess morphology and pluripotency. *Top panel:* Example images of three classified colony types: round, AP staining positive (pluripotent); flat, AP staining negative (differentiated); dispersed, AP staining negative (differentiated). Scale bar = 100 μm. *Bottom panel:* bar charts of the mean percentages of ESC colony morphologies for PRC1$^{CKO}$ cells, and the same cells exogenously expressing RING1B-HT or RING1B$^{NBM}$-HT. Error bars indicate SEM from *n* = 3 biological replicates with at least 100 colonies classified each. Means for each replicate are represented by black dots. Source data are provided as a Source Data file.
- **b** Western blot showing exogenous expression of RPCD-HaloTag using a FLAG epitope included in the expressed fusion protein. HDAC1 is included as a loading control. *n* = 1 biological replicate. Source data are provided as a Source Data file.

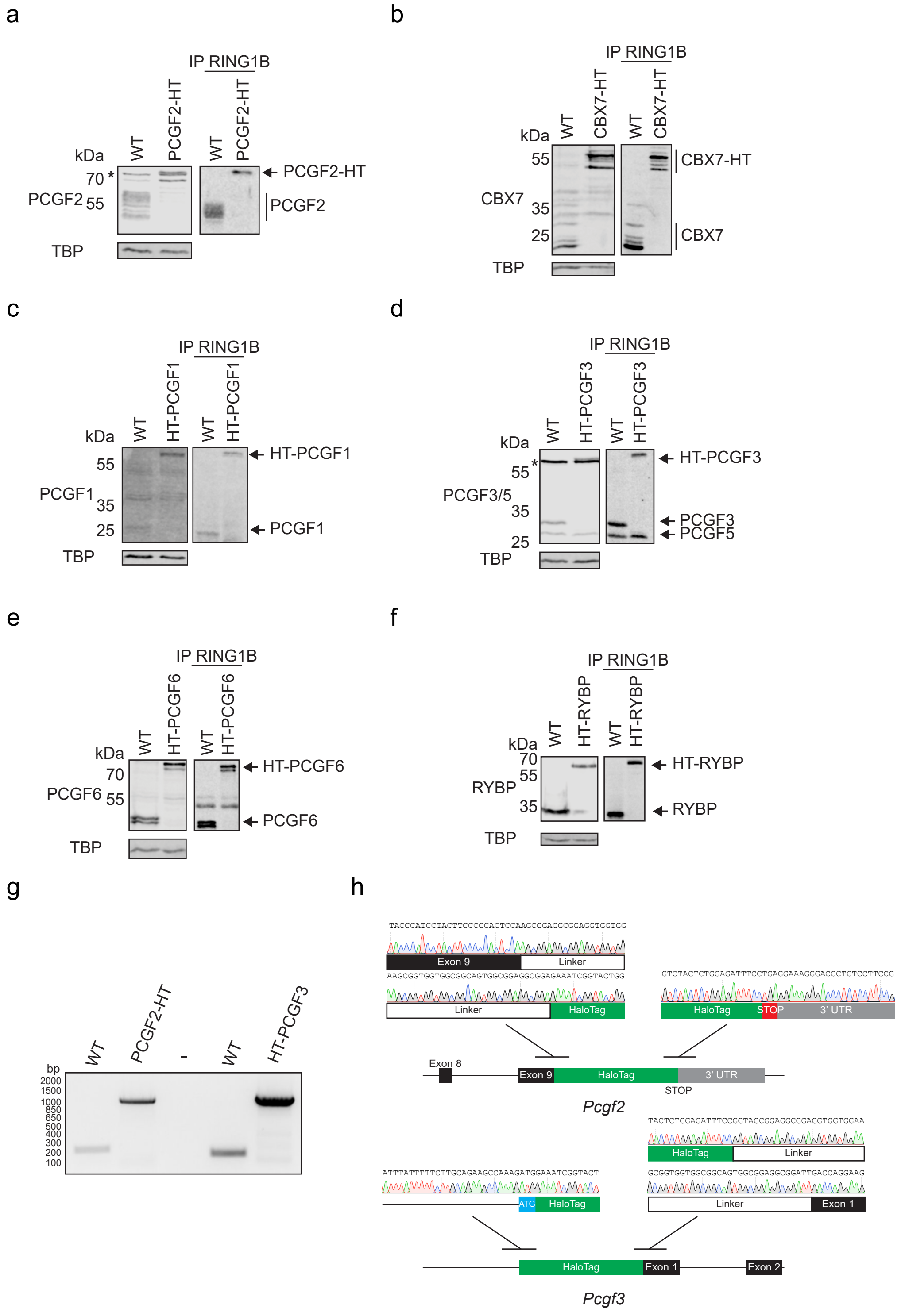


Supplementary Fig. 4

**Supplementary Fig. 4 – HaloTag fusions of PRC1 subunits are expressed and interact with the other complex components similarly to wild type proteins.**

- **a - f** Western blots showing nuclear extracts (left panels) and RING1B immunoprecipitations (right panels) demonstrating homozygous tagging of PRC1 subunits and normal PRC1 complex formation. TBP is included as a loading control for nuclear extracts. Nonspecific bands in a and d are indicated by *. The expected size shift caused by addition of the HaloTag and linker is 35 kDa. $n$ = 1 biological replicate. Source data are provided as a Source Data file.
- **g** An ethidium bromide-stained agarose gel showing PCR products from amplification across insertion sites for the HaloTag in the PCGF2-HaloTag cell line (left) and HaloTag-PCGF3 cell line (right), and the corresponding PCR product in untagged cells (WT) to confirm homozygous tagging. $n$ = 1 biological replicate. Source data are provided as a Source Data file.
- **h** Schematics of HaloTag insertion sites in PCGF2 and PCGF3 cell lines showing sequencing traces across the insertion boundaries.

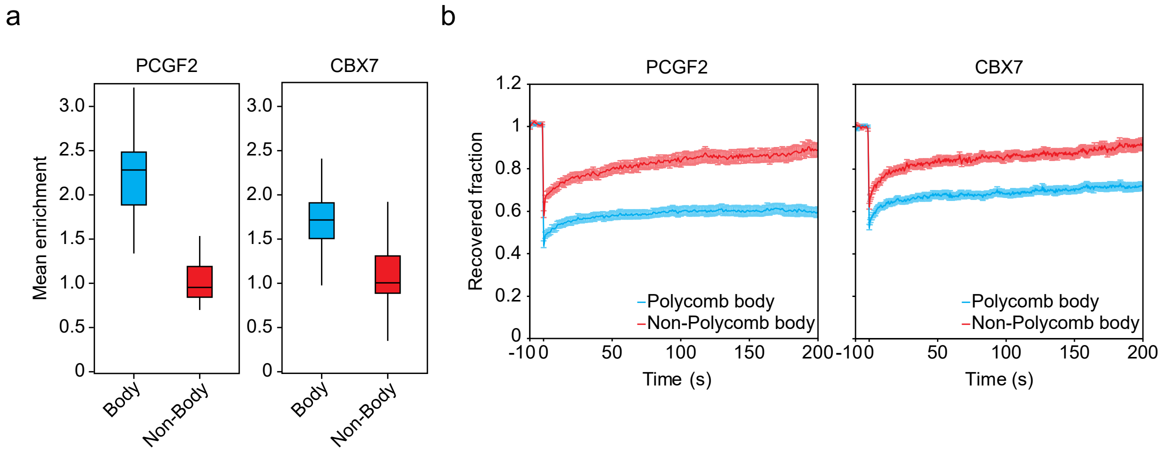


Supplementary Fig. 5

**Supplementary Fig. 5 – Canonical PRC1 subunits are more enriched in Polycomb bodies where they exhibit stable binding.**

**a** Box plots illustrating the relative mean fluorescence signal per unit volume of all PCGF2 and CBX7 Polycomb bodies in each cell (Body, blue) compared to the remaining nuclear volume in the same cell (Non-Body, red), normalised to the median non-Polycomb body fluorescence. *n* = 36 cells for PCGF2, *n* = 57 cells for CBX7, each from 2 experiments. Source data are provided as a Source Data file.

**b** FRAP recovery curves for PCGF2-HT and CBX7-HT in regions containing a Polycomb body (blue) or elsewhere in the nucleus (Non-Polycomb body, red). The recovered fraction was measured relative to initial fluorescence intensity, corrected using an unbleached region. Error bars denote SEM for *n* > 30 regions per condition from 2 experiments each. Source data are provided as a Source Data file.

In **a – b**, boxes represent the interquartile range (IQR), the middle line corresponds to the median, and whiskers extend to the largest and smallest values no more than 1.5 x IQR from the box. Values outside of this range are not plotted, but are included in all analyses.

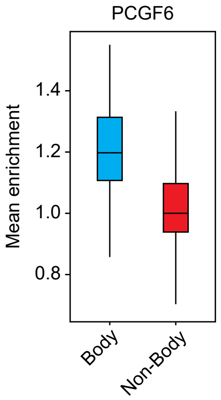


Supplementary Fig. 6

**Supplementary Fig. 6 – PCGF6 is only weakly enriched in Polycomb bodies.**

A box plot illustrating the relative mean fluorescence signal per unit volume of all PCGF6 Polycomb bodies in each cell (Body, blue) compared to the remaining nuclear volume in the same cell (Non-Body, red), normalised to the median non-Polycomb body fluorescence. *n* = 44 cells from 2 experiments. Boxes represent the interquartile range (IQR), the middle line corresponds to the median, and whiskers extend to the largest and smallest values no more than 1.5 x IQR from the box. Values outside of this range are not plotted, but are included in all analyses. Source data are provided as a Source Data file.

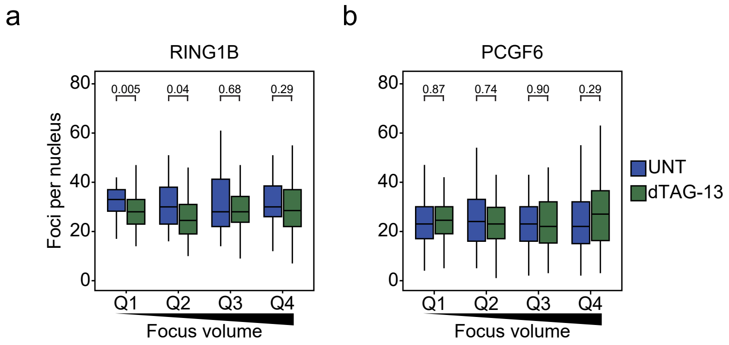


Supplementary Fig. 7

**Supplementary Fig. 7 – Removal of PRC2 and H3K27me3 has a small effect on Polycomb body volumes observed for RING1B and PCGF6.**

**a** A box plot comparing numbers of nuclear foci counted for RING1B with (green) and without (blue) 96 hr dTAG-13 treatment, with foci divided into quartiles based on focus volumes in untreated cells. $n > 50$ cells per condition from 2 experiments. Indicated *p*-values were calculated using a two-tailed Student's *t* test. Source data are provided as a Source Data file.

**b** As in **a**, for PCGF6. Source data are provided as a Source Data file.

In **a – b**, boxes represent the interquartile range (IQR), the middle line corresponds to the median, and whiskers extend to the largest and smallest values no more than 1.5 x IQR from the box. Values outside of this range are not plotted, but are included in all analyses.

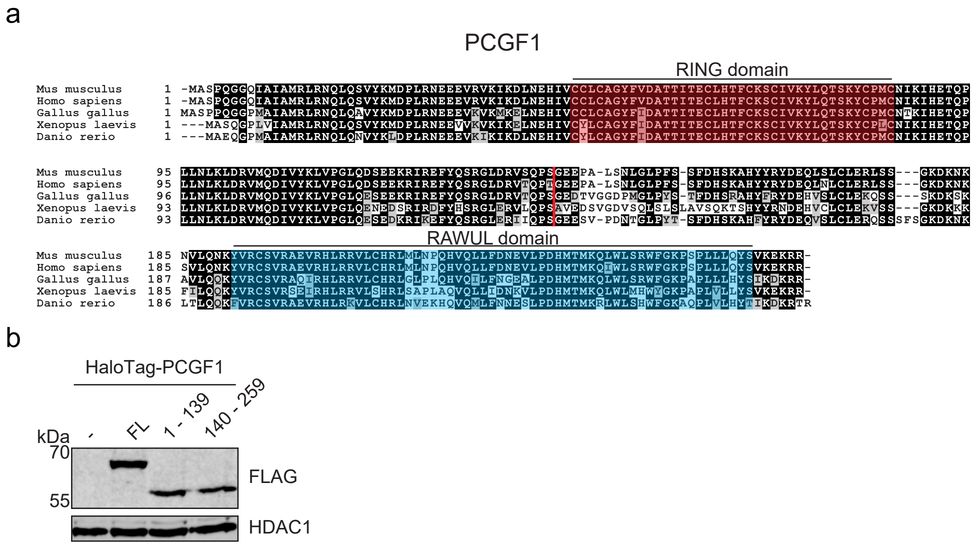


Supplementary Fig. 8

**Supplementary Fig. 8 – PCGF1 can be separated into two fragments that correspond to evolutionarily conserved domains.**

- **a** Alignments of PCGF1 amino acid sequences across vertebrate organisms produced by Clustal Omega[1]. Residues shaded in black are identical across 50% of sequences. Residues which are similar are shaded in grey. Positions of RING (red) and RAWUL (blue) domains in the *Mus musculus* sequence are annotated. Red line indicates the site at which the amino acid sequence was split to generate N- and C-terminal truncations.
- **b** Western blot showing exogenous expression of HaloTag fusions of full length PCGF1 (FL) and N- (1 – 139) and C-terminal (140 – 259) truncations, using a FLAG epitope included in the expressed fusion protein. HDAC1 is included as a loading control. *n* = 1 biological replicate. Source data are provided as a Source Data file.

**Supplementary Table 1 – List of primers.**

| Primer | Sequence |
|---|---|
| RING1B-HT HA 1 Fwd | GAAGGAAGGCCGTCAAGGCCGCATGGTTTTTTTGGCTCCATGATTTAGGAA C |
| RING1B-HT HA 1 Rev | CGCTTCCACCACCTCCGCCTCCGCTTTTGTGCTCCTTGGTGGGT |
| RING1B-HT Tag Fwd | TTATGCACCCACCAAGGAGCACAAAAGCGGAGGCGGAGG |
| RING1B-HT Tag Rev | CTTAGAATTGACTTTTAAAAGCTCAACCGGAAATCTCCAGAGTAGACAG |
| RING1B-HT HA 2 Fwd | GCTGTCTACTCTGGAGATTTCCGGTTGAGCTTTTAAAAGTCAATTCTAAGAC TGAAC |
| RING1B-HT HA 2 Rev | GAAAGGAAGGCCCATGAGGCCCAGAGAAATGAGGGGAAGCTTTGAACA |
| RING1B-HT Screen Fwd | CTCACTGTTACTGAGCACCA |
| RING1B-HT Screen Rev | TGAACATATCCCAGGTCTGTCC |
| H2B-HT assembly Fwd | CGGAAGCGGTGGAGGCGGAGCTAGCGAAATCGGTACTGGCTTTCCATTCG |
| H2B-HT assembly Rev | TAACTCGACTCTAGGCGGCCGCCTAGGAAATCTCCAGAGTAGACAGCCAG |
| RPCD-HT assembly 1 Fwd | TCATTTTGGCAAAGAATTCCTCGAGGCCACCATGGGTATGTCTCAGGCTGT GCAGAC |
| RPCD-HT assembly 1 Rev | CCTCCGCTCTGGCCATTGGCAGCATC |
| RPCD-HT assembly 2 Fwd | TGGCCAGAGCGGAGGCGGAGG |
| RPCD-HT assembly 2 Rev | CCTCCTCCACCGGAAATCTCCAGAGTAGACAG |
| RPCD-HT assembly 3 Fwd | TTCCGGTGGAGGAGGAAGCGATTATAAAGATGATGAT |
| RPCD-HT assembly 3 Rev | CGAGGCTGATCAGCGGATCCTTAAACCTTTCTCTTCTTTTTTGGAACCTTTCT CTTCTTTTTTGGAA |
| RING1B-HT TIGRE assembly 1 Fwd | TCATTTTGGCAAAGAATTCCTCGAGGCCACCATGGGTATGTCTCAGGCTGT GCAGAC |
| RING1B-HT TIGRE assembly 1 Rev | CCTCCGCTTTTGTGCTCCTTGGTGGGTG |
| RING1B-HT TIGRE assembly 2 Fwd | GCACAAAAGCGGAGGCGGAGG |
| RING1B-HT TIGRE assembly 2 Rev | CCTCCTCCACCGGAAATCTCCAGAGTAGACAG |
| RING1B-HT TIGRE assembly 2 Fwd | TTCCGGTGGAGGAGGAAGCGATTATAAAGATGATGAT |
| RING1B-HT TIGRE assembly 2 Rev | CGAGGCTGATCAGCGGATCCTTACCCGGGTGATCTAGCTCCT |

| | |
|---|---|
| RING1B NBM Mutagenesis Round 1 Fwd | GATTGTATTATCACAGCCCTTGCAAGTGGCAACAAAGAGTGTC |
| RING1B NBM Mutagenesis Round 1 Rev | GACACTCTTTGTTGCCACTTGCAAGGGCTGTGATAATACAATC |
| RING1B NBM Mutagenesis Round 2 Fwd | GTTCGGGTCTGGCCTTAGTGATGCTGCAGAAACCAGTGCTTTCCGACAGGTAGGACACTCTT |
| RING1B NBM Mutagenesis Round 2 Rev | AAGAGTGTCCTACCTGTCGGAAAGCACTGGTTTCTGCAGCATCACTAAGGCCAGACCCGAAC |
| TIGRE no insert screen Fwd | CCAGTCAGGCACAAGAGGTC |
| TIGRE no insert screen Rev | GCCAGGACCCACAAAGTGAA |
| TIGRE insert screen Fwd | CAGAGCGCGTCAAAGGTATT |
| TIGRE insert screen Rev 1 | GAGGCGGTGCTTAGCC |
| TIGRE insert screen Rev 2 | AACCGAGGCGCTTTGTG |
| Strep-His-RING1B Fwd | TTTAACTTTAAGAAGGAGATATACAATGGCAAGCTGGAGCC |
| Strep-His-RING1B Rev | CGGAGCTCGAATTCGTCATTTGTGCTCCTTGGTGGGTG |
| Strep-His-RING1B-HT 1 Rev | CCTCCGCTTTTGTGCTCCTTGGTGGGTG |
| Strep-His-RING1B-HT 2 Fwd | GCACAAAAGCGGAGGCGGAGG |
| Strep-His-RING1B-HT 2 Rev | CGGAGCTCGAATTCGTCAACCGGAAATCTCCAGAGTAGACAG |
| HT-PCGF1 HA 1 Fwd | GAAGGAAGGCCGTCAAGGCCGCATGCCTCTCGTGCATCCATTTGT |
| HT-PCGF1 HA 1 Rev | CGAATGGAAAGCCAGTACCGATTTCCATCGCGATCGCAATCTGGC |
| HT-PCGF1 Tag Fwd | GGGGGGCCAGATTGCGATCGCGATGGAAATCGGTACTGGCTTTCCATTCG |
| HT-PCGF1 Tag Rev | CTGACTGGAGCTGGTTCCGAAGCCTTCCGCCTCCGCCACT |
| HT-PCGF1 HA 2 Fwd | TGGTGGCGGCAGTGGCGGAGGCGGAAGGCTTCGGAACCAGCTCC |
| HT-PCGF1 HA 2 Rev | GAAAGGAAGGCCCATGAGGCCCAGACTAAGGAGGGGTGAAGGGC |
| HT-PCGF1 Screen Fwd | CAATCCGGAGAATGTGAGGC |
| HT-PCGF1 Screen Rev | GGTTCACACAGCTAACGGAC |
| PCGF2-HT HA 1 Fwd | CGTCAAGGCCGCATGCCACCTTTCAATTCACTTACCTCTATCCCC |
| PCGF2-HT HA 1 Rev | CCTCCGCTTGGAGTGGGGGAAGTAGGATGG |
| PCGF2-HT Tag Fwd | CACTCCAAGCGGAGGCGGAGG |

| | |
|---|---|
| PCGF2-HT Tag Rev | CCTTTCCTCAGGAAATCTCCAGAGTAGACAGCCAG |
| PCGF2-HT HA 2 Fwd | GAGATTTCCTGAGGAAAGGGACCCTCTCCTTC |
| PCGF2-HT HA 2 Rev | CCCATGAGGCCCAGAGACCCACTAGTCAGCTAGGGC |
| PCGF2-HT Screen Fwd | AGGAGGGAAAAGATGGCTGC |
| PCGF2-HT Screen Rev | CCCCAGCTACCCATCCTACT |
| HT-PCGF3 HA 1 Fwd | CGTCAAGGCCGCATGGGAGCAAAGCAATATCTAGGTGGTG |
| HT-PCGF3 HA 1 Rev | ACCGATTTCCATCTTTGGCTTCTGCAAGAAAAATAAATACATGG |
| HT-PCGF3 Tag Fwd | CCAAAGATGGAAATCGGTACTGGCTTTCCATTCG |
| HT-PCGF3 Tag Rev | CTGGTCAATCCGCCTCCGCCAC |
| HT-PCGF3 HA 2 Fwd | AGGCGGATTGACCAGGAAGATTAAACTCTGGGATATAAATG |
| HT-PCGF3 HA 2 Rev | CCCATGAGGCCCAGAGGACTGGCGAAAGCAAAACTGT |
| HT-PCGF3 Screen Fwd | GCAGCCCAGATCAGTCATCA |
| HT-PCGF3 Screen Rev | AGTCACTGTGGTTGCGTCAA |
| HT-PCGF6 HA 1 Fwd | CGTCAAGGCCGCATGGCATGTTTTGCTTGCATATATGTATGTTCAC |
| HT-PCGF6 HA 1 Rev | CCGATTTCCATGGCGGGGATCAGAAAT |
| HT-PCGF6 Tag Fwd | CGCCATGGAAATCGGTACTGGCTTTCCATTCG |
| HT-PCGF6 Tag Rev | GCCTCGTCTCCGCCTCCGCCACTG |
| HT-PCGF6 HA 2 Fwd | AGGCGGAGACGAGGCTGAGACGGACG |
| HT-PCGF6 HA 2 Rev | CCCATGAGGCCCAGAGACATATAGGAAACTAACAGTGAAAGGCACCTGT |
| HT-PCGF6 Screen Fwd | CTTGTTTTCCGTGGCGTCC |
| HT-PCGF6 Screen Rev | AGGTAGCCTAGGTGAACCACA |
| CBX7-HT HA 1 Fwd | GAAGGAAGGCCGTCAAGGCCGCATGCCACGTTTGAGACCCAGAGA |
| CBX7-HT HA 1 Rev | CGCTTCCACCACCTCCGCCTCCGCTCAGCTTCTCGTTGCGGTC |
| CBX7-HT Tag Fwd | CTTCCGAGACCGCAACGAGAAGCTGAGCGGAGGCGGAGG |
| CBX7-HT Tag Rev | TTTTAAGAGGGGAAGCCGCTATTCAACCGGAAATCTCCAGAGTAGACAG |
| CBX7-HT HA 2 Fwd | GCTGTCTACTCTGGAGATTTCCGGTTGAATAGCGGCTTCCCCTC |
| CBX7-HT HA 2 Rev | GAAAGGAAGGCCCATGAGGCCCAGACTGACTGCCCACATACCACT |
| CBX7-HT Screen Fwd | CGCTCCCCTCAAGTGAAGTT |
| CBX7-HT Screen Rev | GCCCCAAGAAGCACAGTTTT |
| HT-RYBP HA 1 Fwd | GAAGGAAGGCCGTCAAGGCCGCATGGTCCTCTTATTGGTTGCCCG |
| HT-RYBP HA 1 Rev | CGAATGGAAAGCCAGTACCGATTTCCATGGACGGGCTGGCCCC |
| HT-RYBP Tag Fwd | GGCGCCGGGGGCCAGCCCGTCCATGGAAATCGGTACTGGCTTTCCATTCG |

| | |
|---|---|
| HT-RYBP Tag Rev | TCGGGCTCTTCTTGTCGCCCATGGTTCCGCCTCCGCCACT |
| HT-RYBP HA 2 Fwd | TGGTGGCGGCAGTGGCGGAGGCGGAACCATGGGCGACAAGAAGAG |
| HT-RYBP HA 2 Rev | CCCATGAGGCCCAGAGCCCCACCTGGTTAAATCTC |
| HT-RYBP Screen Fwd | GACCGCGCTTCTCCTCTG |
| HT-PCGF6 Screen Rev | CTCTTCTTGTCGCCCATGGT |
| HT-SUZ12 HA 1 Fwd | GAAGGAAGGCCGTCAAGGCCGCATGAAGCCACAACCCAATTCCAC |
| HT-SUZ12 HA 1 Rev | ACTCCCATCGCGGTTCCTGC |
| HT-SUZ12 Tag Fwd | AACCGCGATGGGAGTGCAGGTGGAAAC |
| HT-SUZ12 Tag Rev | CCCCACCGCCGTGCTTCTGAGGCGCTCCGCCTCCGCCACT |
| HT-SUZ12 HA 2 Fwd | TGGTGGCGGCAGTGGCGGAGGCGGAGCGCCTCAGAAGCACGG |
| HT-SUZ12 HA 2 Rev | GAAAGGAAGGCCCATGAGGCCCAGAAGGTTTCAAATGCTGCGTGT |
| HT-SUZ12 Screen Fwd | AGCGGTTGGTGTAGCAGG |
| HT-SUZ12 Screen Rev | CTTCTTCACCGGCAACACC |
| HT-PCGF1 TIGRE 1 Fwd | TCATTTTGGCAAAGAATTCCTCGAGGCCACCATGGATTATAAAGATGATGAT GATAAAGG |
| HT-PCGF1 TIGRE 1 Rev | TACCGATTTCAACCTTTCTCTTCTTTTTTGGAACCTTTCTC |
| HT-PCGF1 TIGRE 2 Fwd | GAGAAAGGTTGAAATCGGTACTGGCTTTCCATTCG |
| HT-PCGF1 TIGRE 2 Rev | GGAGACGCTCCGCCTCCGCCACTG |
| HT-PCGF1 TIGRE 3 Fwd | AGGCGGAGCGTCTCCTCAGGGGGG |
| HT-PCGF1 TIGRE 3 Rev | CGAGGCTGATCAGCGGATCCCTACCTCCTCTTCTCTTTCACACTGTATTGGA G |
| HT-PCGF1 1-139 TIGRE 3 Rev | CGAGGCTGATCAGCGGATCCCTAACTGGGCTGGGAGACTCTG |
| HT-PCGF1 140-259 TIGRE 2 Rev | TCTTCACCTCCGCCTCCGCCACTG |
| HT-PCGF1 140-259 TIGRE 3 Fwd | AGGCGGAGGTGAAGAGCCAGCCCT |

## Supplementary Table 2 – List of sgRNA sequences.

| **sgRNA target** | **Sequence** |
|---|---|
| *Ring1b* | GCTCATTTGTGCTCCTTGGT |
| *Pcgf1* | GCCTCATCGCGATCGCAATC |
| *Pcgf2* | CCCTTTCCTCAAGGGGGGCA |
| *Pcgf3* | ATCTTCCTGGTCAACATCTT |
| *Pcgf6* | TCTGATCCCCGCCATGGACG |
| *Cbx7* | CTATTCACAGCTTCTCGTTG |
| *Rybp* | GCCCATGGTCATGGACGGGC |
| *Suz12* | GTGCTTCTGAGGCGCCATCG |
| TIGRE locus | ACTGCCATAACACCTAACTT |

**Supplementary Table 3 – List of antibodies.**

| Antibody | Source or reference | Identifiers | Dilution or quantity |
|---|---|---|---|
| Rabbit monoclonal anti-H2AK119ub1 | Cell Signaling Technology | Cat# D27C4 | 1:2000 |
| Rabbit monoclonal anti-H3K27me3 | Cell Signaling Technology | Cat# C36B11 | 1:2000 |
| Rabbit monoclonal anti-RING1B (WB) | Cell Signaling Technology | Cat# D22F2 | 1:2000 |
| Mouse monoclonal anti-RING1B (IP) | Atsuta et al., 2001[2] | | 3 µg |
| Rabbit monoclonal anti-SUZ12 | Cell Signaling Technology | Cat# D39F6 | 1:1000 |
| Mouse monoclonal anti-H3 | Cell Signaling Technology | Cat# 96C10 | 1:1000 |
| Rabbit polyclonal anti-PCGF6 | LifeSpan BioSciences | Cat# LS-C482495 | 1:1000 |
| Rabbit polyclonal anti-PCGF2 (Mel-18) | Santa Cruz | Cat# sc-10744 | 1:1000 |
| Rabbit polyclonal anti-PCGF1 | In house[3] (via PTUBS) | | 1:1000 |
| Rabbit monoclonal anti-PCGF3+PCGF5 | Abcam | Cat# ab201510 | 1:1000 |
| Rabbit polyclonal anti-CBX7 | Millipore | Cat# 07-981 | 1:1000 |
| Rabbit monoclonal anti-BRG1 | Abcam | Cat# ab110641 | 1:3000 |
| Mouse monoclonal anti-TBP | Abcam | Cat# ab818 | 1:3000 |
| Mouse monoclonal anti-FLAG | Sigma | Cat# F1804 | 1:1000 |
| Goat polyclonal anti-Mouse Alexa Fluor 488-conjugated | Invitrogen | Cat# A-11029 | 1:10000 |
| IRDye 800CW Goat anti-Mouse IgG | LI-COR | Cat# 926-32210 | 1:15000 |
| IRDye 800CW Goat anti-Rabbit IgG | LI-COR | Cat# 926-32211 | 1:15000 |
| IRDye 680RD Goat anti-Mouse IgG | LI-COR | Cat# 926-68070 | 1:15000 |
| IRDye 680RD Goat anti-Rabbit IgG | LI-COR | Cat# 926-68071 | 1:15000 |

**Supplementary Table 4 – Summary of calculated numbers of molecules, bound fractions, fractions of observed binding events which were stable, and stable binding times for this study.**

Values are given with +/- standard deviation (numbers of molecules) or SEM (bound fraction, stable fraction, stable binding times) where appropriate. Nuclear volume used to calculate nuclear concentration was determined from segmented nuclei as 920 μm3.

| **Protein** | **Molecules per cell** | | **Nuclear concentration (nM)** | **Bound fraction** | **Stable fraction of observed binding events** | **Stable binding time (s)** |
|---|---|---|---|---|---|---|
| | **In-gel fluorescence** | **Fluorescence microscopy** | | | | |
| RING1B | 63200 +/- 8400 | standard | 113 | 0.20 +/- 0.01 | 0.38 +/- 0.02 | > 100 |
| PCGF1 | 5200 +/- 3600 | 7200 +/- 1400 | 9 | 0.26 +/- 0.01 | 0.44 +/- 0.03 | > 100 |
| PCGF2 | 25600 +/- 4800 | 33500 +/- 8800 | 46 | 0.18 +/- 0.007 | 0.44 +/- 0.02 | > 100 |
| PCGF3 | 2800 +/- 1500 | 3500 +/- 900 | 5 | 0.13 +/- 0.008 | 0.28 +/- 0.05 | 40 +/- 10 |
| PCGF6 | 14000 +/- 2800 | 15800 +/- 3100 | 25 | 0.16 +/- 0.009 | 0.35 +/- 0.04 | 50 +/- 20 |
| CBX7 | 19000 +/- 2800 | 18800 +/- 5800 | 34 | 0.18 +/- 0.007 | 0.50 +/- 0.03 | > 100 |
| RYBP | 29600 +/- 4500 | 28500 +/- 8300 | 53 | 0.13 +/- 0.008 | 0.44 +/- 0.03 | 30 +/- 5 |

**Supplementary Table 5 – Numbers of molecules tracked on average per video listed by protein.**

Values determined as means of all videos from each experiment, counting tracks with >4 localisations for 67 Hz experiments and >1 localisation for 2 Hz and 0.033 Hz experiments. ND = not done.

| Protein | Mean number of tracked molecules | | |
|---|---|---|---|
| | 67 Hz tracks | 2 Hz tracks | 0.033 Hz tracks |
| H2B | 195 | - | ND |
| HaloTag-3xNLS | 1080 | ND | ND |
| RING1B | 617 | 56 | 73 |
| RING1B (TIGRE) | 121 | 36 | ND |
| RING1B NBM (TIGRE) | 147 | 51 | ND |
| RPCD | 199 | ND | ND |
| PCGF1 | 96 | 23 | ND |
| PCGF2 | 212 | 59 | ND |
| PCGF3 | 163 | 12 | ND |
| PCGF6 | 223 | 20 | ND |
| CBX7 | 303 | 36 | ND |
| RYBP | 591 | 47 | ND |
| RING1B (dTAG-SUZ12 UNT) | 254 | 97 | ND |
| RING1B (dTAG-SUZ12 dTAG-13) | 204 | 87 | ND |
| PCGF2 (dTAG-SUZ12 UNT) | 205 | 78 | ND |
| PCGF2 (dTAG-SUZ12 dTAG-13) | 209 | 67 | ND |
| PCGF6 (dTAG-SUZ12 UNT) | 186 | 37 | ND |
| PCGF6 (dTAG-SUZ12 dTAG-13) | 204 | 43 | ND |
| PCGF1 FL (TIGRE) | 175 | 58 | ND |
| PCGF1 1-139 (TIGRE) | 50 | 7 | ND |
| PCGF1 140-259 (TIGRE) | 176 | 113 | ND |

## Supplementary Table 6 – List of genomics datasets

| Dataset | Source | Data series | Sample numbers |
|---|---|---|---|
| RING1B ChIP-seq | Blackledge et al., 2020[4] | GSE132752 | GSM3891386, GSM3891387, GSM3891388 |
| PCGF1 ChIP-seq | Blackledge et al., 2020[4] | GSE132752 | GSM3891362, GSM3891363, GSM3891364 |
| PCGF2 ChIP-seq | Blackledge et al., 2020[4] | GSE132752 | GSM3891368, GSM3891369, GSM3891370 |
| PCGF6 ChIP-seq | Blackledge et al., 2020[4] | GSE132752 | GSM3891374, GSM3891375, GSM3891376 |
| H2AK119ub1 ChIP-seq | Blackledge et al., 2020[4] | GSE132752 | GSM3891325, GSM3891326, GSM3891327 |
| CBX7 ChIP-seq | Fursova et al., 2019[5] | GSE119618 | GSM3581143, GSM3581144, GSM3581145 |

**Supplementary References**


1. Madeira, F. *et al.* The EMBL-EBI search and sequence analysis tools APIs in 2019. *Nucleic Acids Res.* **47**, W636–W641 (2019).
2. Atsuta, T. *et al.* Production of Monoclonal Antibodies Against Mammalian Ring1B Proteins. *Hybridoma* **20**, 43–46 (2001).
3. Blackledge, N. P. *et al.* Variant PRC1 complex-dependent H2A ubiquitylation drives PRC2 recruitment and polycomb domain formation. *Cell* **157**, 1445–1459 (2014).
4. Blackledge, N. P. *et al.* PRC1 Catalytic Activity Is Central to Polycomb System Function. *Mol. Cell* **77**, 857-874.e9 (2020).
5. Fursova, N. A. *et al.* Synergy between Variant PRC1 Complexes Defines Polycomb-Mediated Gene Repression. *Mol. Cell* **74**, 1020-1036.e8 (2019).
