# Supplementary material for: Live-cell single particle tracking of PRC1 reveals a highly dynamic system with low target site occupancy
Source: Nat Commun. 2021 Feb 9;12:887. doi: 10.1038/s41467-021-21130-6 (PMC7873255; doi:10.1038/s41467-021-21130-6)
Supplement: Supplementary file 3 — Description of Additional Supplementary Files [file 41467_2021_21130_MOESM3_ESM.pdf]

Description of Additional Supplementary Files

Supplementary Data 1 - Positions, read counts and densities of molecules at all RING1B peaks.

Supplementary Movie 1 – Example 67 Hz SPT video for RING1B-HaloTag.
An example movie to illustrate typical data obtained from a 67 Hz video of RING1B-HaloTag. The movie is cropped to show a specific region containing cells, and shortened to half the length of a typical movie (2000 frames, 30 s). The time after acquisition was initiated is shown on each frame. Scale bar = 5 μm.

Supplementary Movie 2 – Example 67 Hz SPT video for H2B-HaloTag.
An example movie to illustrate typical data obtained from a 67 Hz video of H2B-HaloTag. The movie is cropped to show a specific region containing cells, and shortened to half the length of a typical movie (2000 frames, 30 s). The time after acquisition was initiated is shown on each frame. Scale bar = 5 μm.

Supplementary Movie 3 – Example 67 Hz SPT video for HaloTag-3xNLS.
An example movie to illustrate typical data obtained from a 67 Hz video of HaloTag-3xNLS. The movie is cropped to show a specific region containing cells, and shortened to half the length of a typical movie (2000 frames, 30 s). The time after acquisition was initiated is shown on each frame. Scale bar = 5 μm.

Supplementary Movie 4 – Example 2 Hz SPT video for RING1B-HaloTag.
An example movie to illustrate typical data obtained from a 2 Hz video of RING1B-HaloTag. The movie is cropped to show a specific region containing cells. The time after acquisition was initiated is shown on each frame. Scale bar = 5 μm.

Supplementary Movie 5 – Example 0.033 Hz SPT video for RING1B-HaloTag.
An example movie to illustrate typical data obtained from a 0.033 Hz video of RING1B-HaloTag. The movie is cropped to show a specific region containing cells. The time after acquisition was initiated is shown on each frame. Scale bar = 5 μm.
