# Supplementary material for: Live-cell single particle tracking of PRC1 reveals a highly dynamic system with low target site occupancy
Source: Nat Commun. 2021 Feb 9;12:887. doi: 10.1038/s41467-021-21130-6 (PMC7873255; doi:10.1038/s41467-021-21130-6)
Supplement: Supplementary file 10 — Reporting summary [file 41467_2021_21130_MOESM10_ESM.pdf]

nature research

Corresponding author(s): Robert J. Klose

Last updated by author(s): Dec 17, 2020

Reporting Summary

Nature Research wishes to improve the reproducibility of the work that we publish. This form provides structure for consistency and transparency in reporting. For further information on Nature Research policies, see our Editorial Policies and the Editorial Policy Checklist.

Statistics

For all statistical analyses, confirm that the following items are present in the figure legend, table legend, main text, or Methods section.

n/a	Confirmed	
[ ]	[x]	The exact sample size (n) for each experimental group/condition, given as a discrete number and unit of measurement
[ ]	[x]	A statement on whether measurements were taken from distinct samples or whether the same sample was measured repeatedly
[ ]	[x]	The statistical test(s) used AND whether they are one- or two-sided<br>*Only common tests should be described solely by name; describe more complex techniques in the Methods section.*
[x]	[ ]	A description of all covariates tested
[ ]	[x]	A description of any assumptions or corrections, such as tests of normality and adjustment for multiple comparisons
[ ]	[x]	A full description of the statistical parameters including central tendency (e.g. means) or other basic estimates (e.g. regression coefficient) AND variation (e.g. standard deviation) or associated estimates of uncertainty (e.g. confidence intervals)
[ ]	[x]	For null hypothesis testing, the test statistic (e.g. F, t, r) with confidence intervals, effect sizes, degrees of freedom and P value noted<br>*Give P values as exact values whenever suitable.*
[x]	[ ]	For Bayesian analysis, information on the choice of priors and Markov chain Monte Carlo settings
[x]	[ ]	For hierarchical and complex designs, identification of the appropriate level for tests and full reporting of outcomes
[x]	[ ]	Estimates of effect sizes (e.g. Cohen's d, Pearson's r), indicating how they were calculated

Our web collection on statistics for biologists contains articles on many of the points above.

Software and code

Policy information about availability of computer code

Data collection	Image Studio v5.2 (LI-COR), Typhoon FLA 7000 Control Software 1.2 (Fujifilm), Volocity v5.4 (PerkinElmer), Andor Solis (Andor Technology)
Data analysis	MATLAB R2013b and R2017b (MathWorks), ImageJ 1.52p through Fiji (Schindelin et al., 2012), Stormtracker v2018 (Uphoff et al., 2013), MACS2 v2.1.1 (Zhang et al., 2008), deepTools v3.0.1 (Ramirez et al., 2014), TANGO v0.99 (Ollion et al., 2013), cellSens v3.1 (Olympus), Spot-On v0.11.5a (Hansen et al., 2018), R v3.6.2 (R Core Team 2019). Polycomb body analysis scripts are available at [https://github.com/MKHuseyin/Polycomb-Body-Analysis].

For manuscripts utilizing custom algorithms or software that are central to the research but not yet described in published literature, software must be made available to editors and reviewers. We strongly encourage code deposition in a community repository (e.g. GitHub). See the Nature Research guidelines for submitting code & software for further information.

Data

Policy information about availability of data

All manuscripts must include a data availability statement. This statement should provide the following information, where applicable:
- Accession codes, unique identifiers, or web links for publicly available datasets
- A list of figures that have associated raw data
- A description of any restrictions on data availability

The ChIP-seq datasets analysed in this current study are available in the GEO repository, with the accession codes GSE132752 and GSE119618. Read counts at RING1B peaks from the ChIP-seq datasets used are available in Table S3.

Field-specific reporting

Please select the one below that is the best fit for your research. If you are not sure, read the appropriate sections before making your selection.

☒ Life sciences ☐ Behavioural & social sciences ☐ Ecological, evolutionary & environmental sciences

For a reference copy of the document with all sections, see nature.com/documents/nr-reporting-summary-flat.pdf

Life sciences study design

All studies must disclose on these points even when the disclosure is negative.

Sample size	Sample sizes were determined based on estimates from preliminary experiments in order to obtain sufficient numbers of molecules (in single molecule imaging experiments) or cells (in whole cell-based methods, such as FRAP, Polycomb body analysis) per imaging experiment to allow for robust processing with analysis software and statistical analysis (for similar studies see e.g. Rhodes et al., Scc2/Nipbl hops between chromosomal cohesin rings after loading, eLife (2017), Hansen et al., Robust model-based analysis of single-particle tracking experiments with Spot-On, eLife (2018).)
Data exclusions	No data were excluded.
Replication	Quantified experimental findings reported in this study were reliably reproduced in multiple biological replicates. The numbers of cells (FRAP and Polycomb body quantification, other cellular imaging approaches), movies (single particle tracking), or experimental replicates (protein quantification, ChIP-seq analysis, western blotting, other aggregate analyses) are given in the figure legends.
Randomization	Randomization was not relevant to this study. In all experiments the genotypes of cell lines being studied were known, and were therefore used to allocate samples into experimental groups. Randomization was therefore not appropriate or relevant.
Blinding	Samples were defined based on the originating cell line and there was no prior knowledge for results, therefore blinding was not performed or relevant to data collection or analysis.

Reporting for specific materials, systems and methods

We require information from authors about some types of materials, experimental systems and methods used in many studies. Here, indicate whether each material, system or method listed is relevant to your study. If you are not sure if a list item applies to your research, read the appropriate section before selecting a response.

Materials & experimental systems

n/a	Involved in the study	
☐	☒	Antibodies
☐	☒	Eukaryotic cell lines
☒	☐	Palaeontology and archaeology
☒	☐	Animals and other organisms
☒	☐	Human research participants
☒	☐	Clinical data
☒	☐	Dual use research of concern

Methods

n/a	Involved in the study	
☒	☐	ChIP-seq
☒	☐	Flow cytometry
☒	☐	MRI-based neuroimaging

Antibodies

Antibodies used

Rabbit monoclonal anti-H2AK119ub1, Cell Signaling Technology, Cat# D27C4
Rabbit monoclonal anti-H3K27me3, Cell Signaling Technology, Cat# C36B11
Rabbit monoclonal anti-RING1B (WB), Cell Signaling Technology, Cat# D22F2
Mouse monoclonal anti-RING1B (IP), Atsuta et al., 2001
Rabbit monoclonal anti-SUZ12, Cell Signaling Technology, Cat# D39F6
Mouse monoclonal anti-H3, Cell Signaling Technology, Cat# 96C10
Rabbit polyclonal anti-PCGF6, LifeSpan BioSciences, Cat# LS-C482495, lot 101287
Rabbit polyclonal anti-PCGF2, Santa Cruz, Cat# sc-10744
Rabbit polyclonal anti-PCGF1, Klose lab purified and characterised (generated by PTUBS) - Blackledge et al., 2014
Rabbit monoclonal anti-PCGF3+PCGF5, Abcam, Cat# ab201510, lot GR291301-2, clone number EPR19475
Rabbit polyclonal anti-CBX7, Millipore, Cat# 07-981, lot 2989489
Rabbit monoclonal anti-BRG1, Abcam, Cat# ab110641, clone number EPNCIR111A
Mouse monoclonal anti-TBP, Abcam, Cat# ab818, clone number 1TBP18
Mouse monoclonal anti-FLAG, Sigma, Cat# F1804, clone number M2
Goat polyclonal anti-Mouse Alexa Fluor 488 conjugated, Invitrogen, Cat# A-11029
IRDye® 800CW Goat anti-Mouse IgG (H + L), LI-COR, Cat# 926-32210

	IRDye® 800CW Goat anti-Rabbit IgG (H + L), LI-COR, Cat# 926-32211<br>IRDye® 680RD Goat anti-Mouse IgG (H + L), LI-COR, Cat# 926-68070<br>IRDye® 680RD Goat anti-Rabbit IgG (H + L), LI-COR, Cat# 926-68071
Validation	anti-H2AK119ub1 - manufacturer-validated against human, mouse, rat and monkey cell lines by western blot<br>anti-H3K27me3 - manufacturer-validated against human, mouse, rat and monkey cell lines by western blot<br>anti-RING1B (WB) - manufacturer-validated against human, mouse, rat and monkey cell lines by western blot, validated here by protein tagging and knockout in mouse cell lines<br>anti-RING1B (IP) - validated in Atsuta et al., 2001<br>anti-SUZ12 - manufacturer-validated against human, mouse, rat and monkey cell lines by western blot, validated here by protein tagging in mouse cell lines<br>anti-H3 - manufacturer-validated against human, mouse, rat and monkey cell lines by western blot<br>anti-PCGF6 - manufacturer-validated by western blot and immunofluorescence for human and mouse cell lines, validated here by protein tagging in mouse cell lines<br>anti-PCGF2 - validated here by protein tagging in mouse cell lines<br>anti-PCGF1 - validated here by protein tagging in mouse cell lines<br>anti-PCGF3+PCGF5 - manufacturer-validated in mouse embryonic stem cells by knockout, validated here by protein tagging in mouse cell lines<br>anti-CBX7 - manufacturer-validated by western blot on mouse embryonic stem cell lysate, validated here by protein tagging in mouse cell lines<br>anti-BRG1 - manufacturer-validated in human, mouse and rat cell lines by western blot and in human cell lines by knockout and IP<br>anti-TBP - manufacturer-validated in U2OS and HeLa cells by ChIP-qPCR and cellular fractionation<br>anti-FLAG - manufacturer-validated in HeLa and CHO cells by western blot and COS-7 cells by immunofluorescence<br>Goat anti-Mouse - manufacturer-validated in human, mouse and Drosophila cells by immunofluorescence<br>IRDye® 800CW Goat anti-Mouse - manufacturer-validated for interaction with mouse IgG, IgM and IgA by ELISA, dot-blot and flow cytometry<br>IRDye® 800CW Goat anti-Rabbit - manufacturer-validated for interaction with rabbit IgG, IgM and IgA by ELISA, dot-blot and flow cytometry<br>IRDye® 680RD Goat anti-Mouse - manufacturer-validated for interaction with mouse IgG, IgM and IgA by ELISA, dot-blot and flow cytometry<br>IRDye® 680RD Goat anti-Rabbit - manufacturer-validated for interaction with rabbit IgG, IgM and IgA by ELISA, dot-blot and flow cytometry

Eukaryotic cell lines

Policy information about cell lines

Cell line source(s)	The PRC1 CKO mouse embryonic stem cell line was previously generated by the Klose lab (Blackledge et al., 2020) and is not available commercially.<br>All other cell lines (listed below) were generated in this study:<br>Mouse ESC: RING1B-HaloTag - this study<br>Mouse ESC: PRC1 CKO TIGRE RING1B-HaloTag - this study<br>Mouse ESC: PRC1 CKO TIGRE RING1B-NBM-HaloTag - this study<br>Mouse ESC: TIGRE RPCD-HaloTag - this study<br>Mouse ESC: HaloTag-PCGF1 - this study<br>Mouse ESC: PCGF2-HaloTag - this study<br>Mouse ESC: HaloTag-PCGF3 - this study<br>Mouse ESC: HaloTag-PCGF6 - this study<br>Mouse ESC: CBX7-HaloTag - this study<br>Mouse ESC: HaloTag-RYBP - this study<br>Mouse ESC: RING1B-HaloTag dTAG-SUZ12 - this study<br>Mouse ESC: PCGF2-HaloTag dTAG-SUZ12 - this study<br>Mouse ESC: HaloTag-PCGF6 dTAG-SUZ12 - this study<br>Mouse ESC: TIGRE HaloTag-PCGF1 - this study<br>Mouse ESC: TIGRE HaloTag-PCGF1 1-139 - this study<br>Mouse ESC: TIGRE HaloTag-PCGF1 140-259 - this study
Authentication	All cell lines generated in this study were validated by PCR, sequencing, and western blotting, and by fluorescence microscopy where appropriate.
Mycoplasma contamination	All cell lines were regularly tested for mycoplasma contamination and were found to be negative.
Commonly misidentified lines<br>(See ICLAC register)	No commonly misidentified cell lines were used.
